# Integrated cytological and transcriptomic analysis reveals insights into pollen fertility in newly synthetic *Brassica* allohexaploids

**DOI:** 10.3389/fpls.2022.1096804

**Published:** 2023-01-13

**Authors:** Zhaoran Tian, Chengyan Ji, Zhengqing Xie, Xinjie Shi, Baoming Tian, Gangqiang Cao, Xiaochun Wei, Yan Yang, Fang Wei, Gongyao Shi

**Affiliations:** ^1^ Henan International Joint Laboratory of Crop Gene Resources and Improvements, School of Agricultural Sciences, Zhengzhou University, Zhengzhou, China; ^2^ Institute of Horticulture, Henan Academy of Agricultural Sciences, Graduate T&R Base of Zhengzhou University, Zhengzhou, China

**Keywords:** anther, *Brassica* allohexaploid, chromosome segregation, pollen fertility, tapetum, meiosis, microspore development, mitosis

## Abstract

Trigenomic *Brassica* allohexaploids (AABBCC, 2n = 6x = 54) have great potential in oilseed breeding and genetic diversity. However, *Brassica* allohexaploids do not exist naturally, and the underlying mechanism regulating pollen fertility in artificially synthesized *Brassica* allohexaploids is still unclear. In this study, synthetic *Brassica* allohexaploids were produced by crossing allotetraploid *B. carinata* (BBCC, 2n = 4x = 34) and diploid *B. rapa* (AA, 2n = 2x = 20), followed by chromosome doubling. The results showed that the pollen fertility was significantly reduced and the pollen structures were mostly distorted, but the nursing anther tapetum developed normally in the synthetic *Brassica* allohexaploids. Furthermore, the data showed that the meiotic events occurred irregularly with uneven chromosome segregation and microspore development appeared mostly abnormal. Transcription analysis showed that the upregulation of genes related to the negative regulation of flower development and the downregulation of genes related to chromosome segregation might play an essential role in reduction of pollen fertility in the *Brassica* allohexaploids. In conclusion, this study elucidated the related mechanisms affecting pollen fertility during male gametophytic development at the cytological and transcriptomic levels in the newly synthesized *Brassica* allohexaploids.

## 1 Introduction

Interspecific hybridization and polyploidization are among the dominant driving forces in plant speciation and evolution (Bennett, 2004). Interspecific hybridization can improve crop quality and yield by transferring favorable traits from distant relatives to synthesized hybrids (Snowdon et al., 2000; Peterka et al., 2004). Polyploidization of hybrids (allopolyploidy) can contribute to phenotypic diversity and adaptation to a wider range of environmental conditions (Sattler et al., 2016). Allopolyploids usually form between relatively closely related species with crossability, either within the same genus or at least within the same tribe (Fitzjohn et al., 2007). This means that there are many regions of sequence similarity in common by descents between the two parental genomes in the new allopolyploids. During meiosis, the presence of these homoeologous regions suggests that the cell machinery often have difficulty in correctly identifying homologous chromosomes in the newly formed allopolyploids.

The pairing homoeologous 1 (Ph1) locus in bread wheat has been identified as controlling diploid-like chromosome pairing by preventing homeologous chromosome pairing (HECP) (Griffiths et al., 2006; Bhullar et al., 2014). In addition, some potential quantitative trait loci for the accurate regulation of meiosis in allopolyploids have only been identified in haploid *B. napus* (Jenczewski et al., 2003) and *Arabidopsis suecica* (Henry et al., 2014; Lloyd and Bomblies, 2016). However, the mechanism regulating the meiotic stability of newly synthesized *Brassica* allohexaploids remains unclear. Compared with the established allopolyploids, the newly synthesized allopolyploids have more synaptic multivalents lasting until metaphase I (MI), resulting in chromosome missegregation, aneuploid gametes and compromised fertility (Szadkowski et al., 2010; Xiong et al., 2011).

Not only meiosis can affect pollen fertility, but abnormal microspore development may lead to decreased pollen fertility. Microgametogenesis includes an asymmetric division to form a big vegetative and a small generative nucleus, and the generative nucleus produces two male gametes after a mitotic division (Huang et al., 2021). In addition, the tapetum is the innermost layer of the four sporophytic layers in the anther wall, which can directly affect the development of gametophytes and is of great importance for the development from microspores to pollen grains (Parish and Li, 2010; Du et al., 2019). As the secretory cell layer, the tapetum provides enzymes for the microspores to be released from the tetrads and nutrients for pollen development.

Allopolyploidy leads to transcriptome reprogramming, and the transcriptome changes of allopolyploidy may be an adaptive mechanism to promote the establishment of gene expression programs and stabilize the evolution of species (Pikaard, 2001). The genus *Brassica* includes six cultivated *Brassica* species, three diploids (*B. rapa*, 2n = 20, AA; *B. nigra*, 2n = 16, BB; *B. oleracea*, 2n = 18, CC) have evolved into three allotetraploids (*B. napus*, 2n = 38, AACC; *B. carinata*, 2n = 34, BBCC; *B. juncea*, 2n = 36, AABB) through pairwise hybridization and subsequent chromosome doubling in natural conditions, termed as “U’s triangle”, which represents a classical evolutionary process and serves as a useful model for polyploidization and chromosomal evolution (Warwick and Al-Shehbaz, 2006). However, there are no *Brassica* allohexaploid species (2n = 54, AABBCC) in nature. Numerous attempts have been made to synthesize *Brassica* allohexaploids through different cross combinations, however, the newly synthesized *Brassica* allohexaploids tend to have lower pollen fertility than the parents (Chen et al., 2020). A series of broad transcriptome changes were produced in *Brassica* allohexaploids compared with their parents, and these changes were gernerally related with the plant growth and development, as well as gamete development (Zhao et al., 2013; Ji et al., 2022).

In the present study, comprehensive cytological and transcriptomic analyses were performed to reveal the underlying mechanism related to pollen fertility in newly formed trigenomic *Brassica* allohexaploids (AABBCC) obtained by hybridization of the allotetraploid *B. carinata* and the diploid *B. rapa*, followed by chromosome doubling. This study provides insights into pollen fertility in newly formed *Brassica* allohexaploids, which will help us in *Brassica* polyploid breeding and genetic improvements in future.

## 2 Materials and methods

### 2.1 Plant materials

The materials used in this study were inbred and kept in our laboratory. The synthetic *Brassica* allohexaploid (AABBCC, 2n = 54) was generated *via* the crossing between the inbreeding line *B. carinata* (2n = 34, BBCC, genotype ‘VI047487’) as the maternal parent and *B. rapa* (2n = 20, AA, genotype ‘JK66-83’) by manual pollination (Yang et al., 2020), following chromosome doubling with colchicine treatment. At 9–12 days after pollination (dap), the immature embryos were rescued on MS agar medium in the growth room. The putatively trigenomic hybrids (ABC, 2n = 27) were treated with colchicine (200 mg/L) for 10 days to double the chromosome numbers on MS agar medium. All the plants were grown in the greenhouse under a 16 h light/8 h dark photoperiod at 22°C.

### 2.2 Chromosome counting and ploidy determination by flow cytometry

The pistils were collected from the developing buds, treated with 2 mM 8-hydroxyquinoline at 20°C for 4 h, then washed with distilled water and fixed in Carnot fixed solution for 4 h. Cytogenetic observation was carried out according to previous study (Miyashita et al., 2011). Fresh young leaves of *B. rapa*, *B. carinata* and *Brassica* allohexaploid plants were taken for ploidy detection. Flow cytometry was performed according to the procedure detailed in previous studies (Yin et al., 2020). The peak fluorescence intensity X-Mean is proportional to the cellular DNA content, so the ploidy of the sample was determined according to the peak position of *B. rapa* and *B. carinata*. Finally, five hexaploid plants (euploid) were confirmed to have 54 chromosomes, so we selected these five *Brassica* allohexaploid plants and five randomly selected *B. rapa* and *B. carinata* respectively for this study.

### 2.3 Pollen grain viability assay

Pollen grain stainability was used as an indication of pollen viability, which was determined by staining at least 3000 pollen grains from randomly selected flower buds of each plant in Alexander staining (Solarbio, Beijing, China) (Alexander, 1969).

### 2.4 Microscopic investigations of anther development after paraffin section

Anthers with determined stages were first fixed in formalin-acetic acid-alcohol (FAA) under a vacuum for 1 h. After dehydration in a graded ethanol series and diaphaneity in a clearing medium (xylene), the samples were embedded in paraffin (Leica, Weztlar, Germany). Sections (8 μm) were obtained with a Leica Reichert Supernova microtome (Leica, Weztlar, Germany), placed on glass slides, and stained with hematoxylin and eosin (Solarbio, Beijing, China) following manufacturer specifications (Park et al., 1998). The sections were examined with a fluorescence microscope (Olympus BX43, Tokyo, Honshu, Japan), and images were captured by a CCD attached camera DP73 (Olympus, Tokyo, Japan) with CellSens Standard software.

### 2.5 Analysis of microspore development

The developing microspores and mature pollens were stained with 4′,6′-diamidino-2-phenylindole (DAPI, Sigma-Aldrich, St. Louis, MO, USA) solution according to the procedures detailed (Oh et al., 2010). After the whole inflorescences were fixed with Carnot solution, the microspores at different development stages were stained with DAPI solution. Using a fluorescence microscope (Olympus BX43, Tokyo, Honshu, Japan) under either bright field or epifluorescence, microscopy imaging was carried out.

### 2.6 Meiotic chromosome behavior observation

Young flowers were harvested and fixed in Carnot fixation solution for 12 h and then transferred to 70% ethanol at 4°C for storage. Meiotic chromosome behavior observations were performed as described previously (Braynen et al., 2017). Meiotic chromosome spreads were prepared as previously described (Zeng et al., 2017). Propidium iodide (PI, Boster Biotechnology, California, USA) solution was applied to stain the prepared slides, and chromosome behavior during meiosis was observed by a fluorescence microscope (Olympus BX43, Tokyo, Honshu, Japan).

### 2.7 Immunofluorescence staining of β-tubulin

For the detection of β-tubulin, procedures from a previous study were adopted, with few modifications (Yang et al., 2019). Fresh inflorescences were collected and fixed immediately in 4% (w/v) paraformaldehyde. The pollen mother cells (PMCs) were first blocked in 5% BSA for 1h. The PMCs were incubated with monoclonal anti-β-tubulin IgG (Sigma-Aldrich, St. Louis, MO, USA) diluted at a 1:100 ratio for 12 h at 4°C in a moist chamber. Then, the PMCs were incubated with a fluorescein isothiocyanate (FITC)-conjugated anti-mouse IgG (Sigma-Aldrich, St. Louis, MO, USA) diluted at a 1:100 ratio for 2 h at 37°C in a dark chamber. Subsequently, a drop of the cell solution was applied to a clean slide after three PBS rinses, and the slide was then stained with 40 g/mL PI. The prepared glass slides were observed and imaged using a Carl Zeiss Confocal Laser Scanning Microscope (LSM 880, Carl Zeiss AG, Oberkochen, Germany).

### 2.8 Genomic *in situ* hybridization (GISH)

Genomic DNA from *B. nigra* was extracted from fresh leaves by the cetyltrimethylammonium bromide (CTAB) method (Springer, 2010). Genomic DNA probes were labeled with Digoxigenin-11-dUTP using a Nick Translation Kit (Roche, Mannheim, Germany). The PMCs were isolated from *Brassica* allohexaploids during the meiosis stage, fixed in Carnot fixation solution for 12 h, and stored in 70% alcohol. Chromosomes were prepared and GISH was performed by following the previous description (Zhou et al., 2016). The slides were observed under fluorescence microscope (Olympus BX43, Tokyo, Honshu, Japan), and Adobe Photoshop CS6 (SAN Jose, California, USA) was employed for the appropriate adjustment of all the images.

### 2.9 Differentially expressed genes (DEGs) and function analysis

The transcriptome sequencing data of flower buds for *Brassica* allohexaploids, *B. rapa* and *B. carinata* were downloaded from the NCBI Gene Expression Omnibus (GEO) with accession numbers GSE201456, GSE193368 and GSE185639, respectively. The reads were then aligned to the *B. rapa* genome sequence using HISAT2 v2.1.0, the reference genome and the annotation file were downloaded from *B. rapa* genome v3.5 sequence (http://Brassicadb.cn, accessed on 23 May 2022) (Kim et al., 2015). Then, the fragments per kilobase per million reads (FPKM) of each gene was calculated to estimate the expression level. DESeq2 was used to perform differential expression analysis on two groups (three biological replicates each), genes with fold change ≥ 2 and false discovery rates (FDR) < 0.05 were designated as DEGs (Love et al., 2014). The Gene Ontology (GO) enrichment analysis was implemented by the hypergeometric test, and GO terms with FDR < 0.05 were considered significantly enriched.

## 3 Results

### 3.1 Reduced pollen fertility in the newly synthesized *Brassica* allohexaploids

To verify the ploidy of the newly synthesized *Brassica* allohexaploid plants, we first performed chromosome counting and flow cytometric analysis of these plants. The mitotic cells of immature pistils in the *B. rapa*, *B. carinata* and *Brassica* allohexaploids showing 20, 34 and 54 chromosomes (**Supplementary Figures S1A–C**). Flow cytometric analysis showed a consistent increase in DNA content in the *Brassica* allohexaploids, indicating potential allohexaploids (**Supplementary Figures S1D–F**). Compared with the parental lines, the possible *Brassica* allohexaploid plants showed vigorous vegetative growth, thickened leaves and larger flowers (**Supplementary Figures S1G–O**). The morphology of *Brassica* allohexaploid plants was similar to that of the maternal line, *B. carinata*, with serrated leaves, yellow flowers and independent vernalization (**Supplementary Figures S1J–O**).

**Figure 1:**
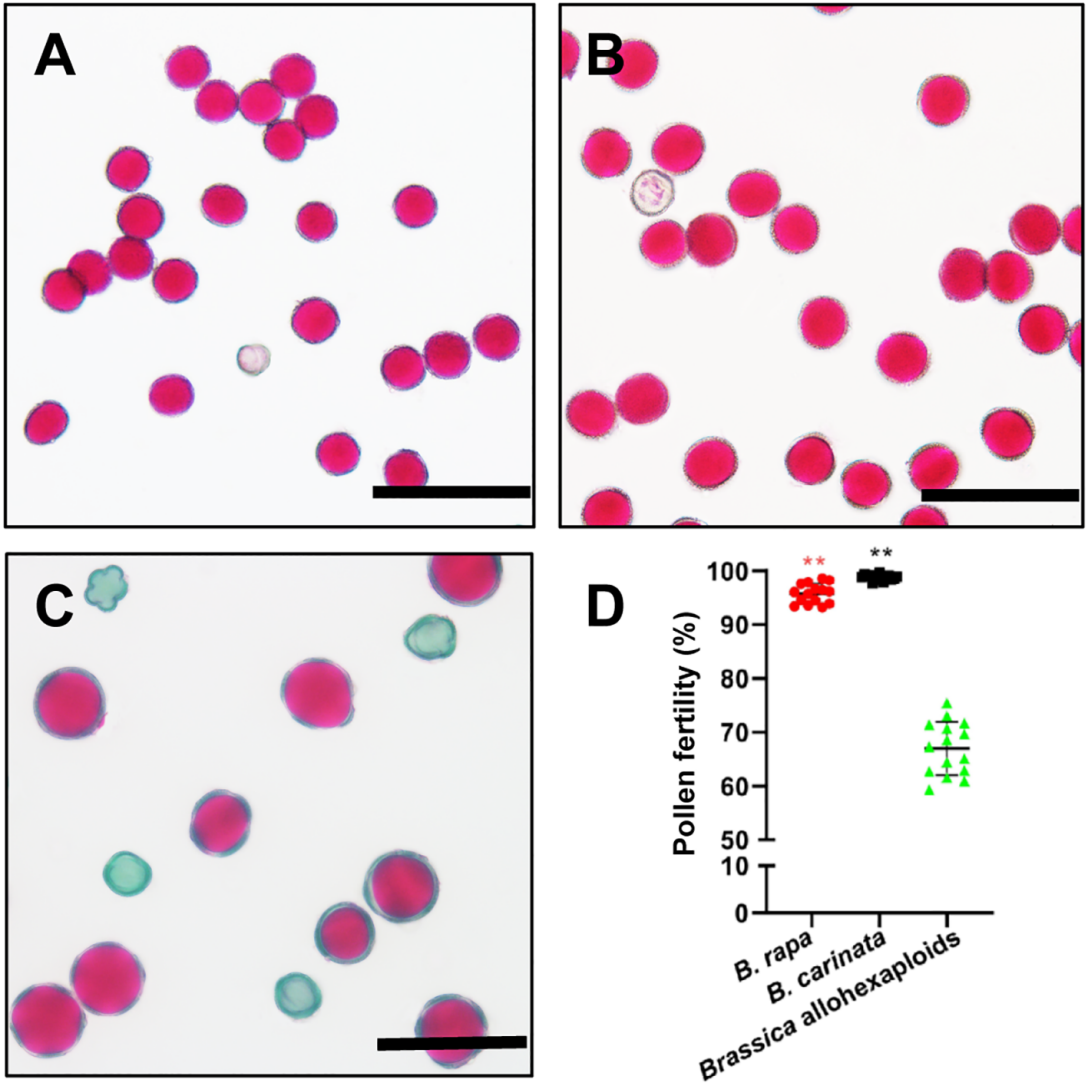
Pollen fertility and morphology observation analyses of *B. rapa*, *B. carinata* and *Brassica* allohexaploids. **(A–C)** Pollen fertility of *B. rapa*, *B. carinata* and *Brassica* allohexaploids determined by Alexander staining. **(D)** Statistical analysis of pollen fertility determined by Alexander staining. ** indicates a significant difference at P ≤ 0.01 analyzed by Analysis of Variance (ANOVA).

The pollen fertility of the *Brassica* allohexaploids was determined by Alexander staining. Compared with both parental lines, the *Brassica* allohexaploids had lower pollen fertility, with an average pollen fertility rate of 66.1% (n =3106), while the average stainable rate of *B. rapa* was 95.8% (n = 3959) and that of *B. carinata* was 98.8% (n = 3795) (**Figure 1**). These results indicated that we had successfully produced the newly synthetic *Brassica* allohexaploid plants with reduced pollen fertility.

### 3.2 Normal anther tapetum development in the *Brassica* allohexaploids

To determine whether tapetum development was affected in the *Brassica* allohexaploids, transverse anther sections were further examined by paraffin sectioning at different stages. No obvious morphological difference in the tapetum was observed between the *Brassica* allohexaploids and the corresponding parents at different anther development stages, and the tapetum, middle layer, endothecium and epidermis all developed normally in the *Brassica* allohexaploids, as well as in the parental lines (**Supplementary Figure S2**). During the microsporogenesis stage, PMCs underwent meiosis and formed tetrads. The nursing tapetum began vacuolating and turned into a secretory cell layer, while the middle layer cells became very thin and tended to degenerate (**Supplementary Figures S2A, E, I**). Subsequently, microspores freely released from the tetrads by callose enzymes produced by the tapetum, which then became more condensed and deeply stained, yet no longer had large vacuoles with hardly visible middle layers (**Supplementary Figures S2B, F, J**). Microspores gradually increased in size and separated into two unequal daughter cells, while the tapetum was further degraded to provide nutrients and materials for microspore development (**Supplementary Figures S2C, G, K**). Although the *Brassica* allohexaploid plants produced some aborted pollen grains at the mature stage, tapetum development and degeneration were normal (**Supplementary Figure S2L**). These observations suggested that the *Brassica* allohexaploids exhibited normal tapetum development and tapetum degeneration to provide nutrients and substances for subsequent gametogenesis.

### 3.3 Defective microspore development in the *Brassica* allohexaploids

**Figure 2:**
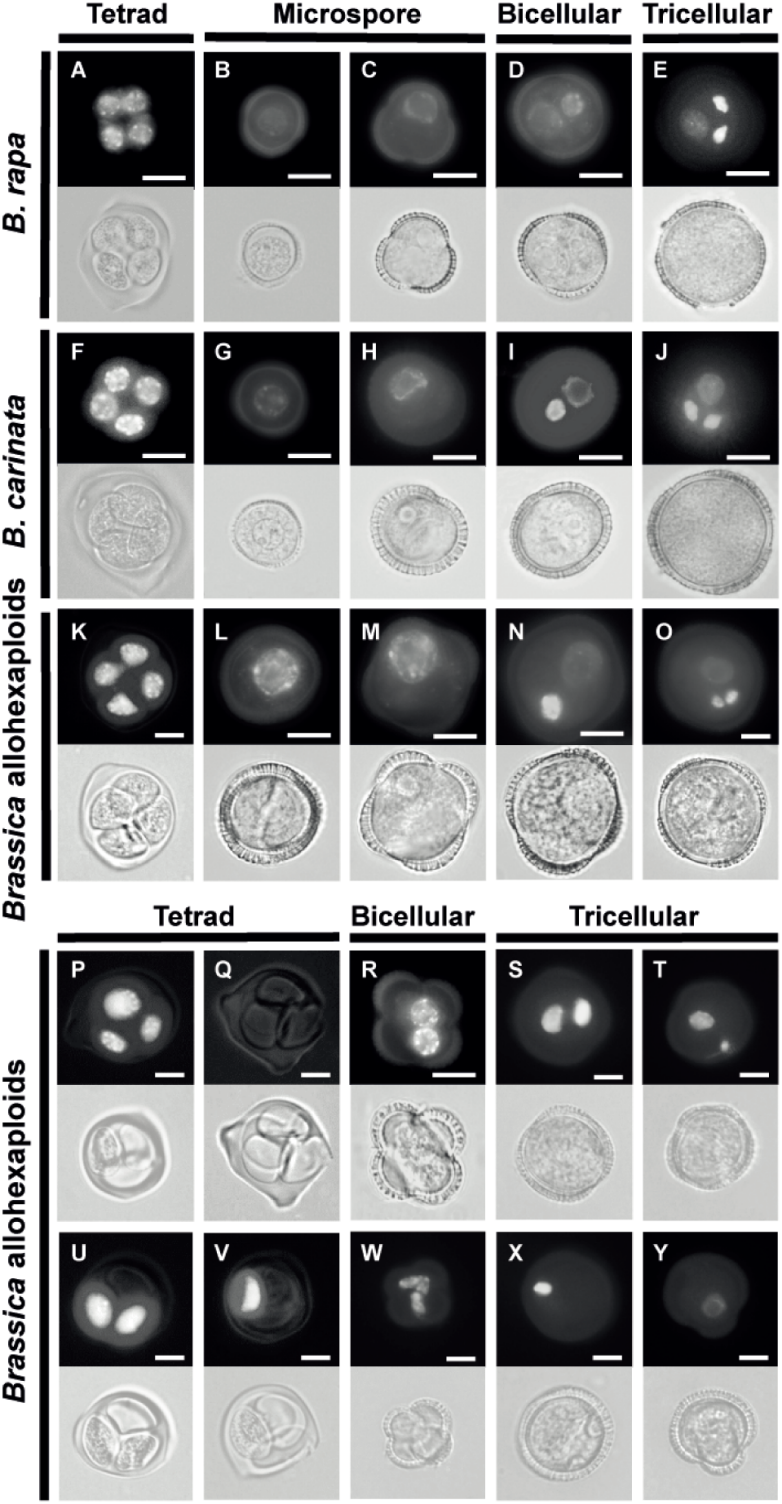
Abnormal pollen development in the synthetic *Brassica* allohexaploids. Normal pollen development in *B. rapa* **(A–E)**, *B. carinata* **(F–J)** and *Brassica* allohexaploids **(K–O)** at different stages. Abnormal pollen development in the *Brassica* allohexaploids at the tetrad stage **(P**, **Q**, **U**, **V)**, the bicellular stage **(R**, **W)** and the tricellular stage **(S**, **T**, **X**, **Y)**. Fluorescent (up) and bright-field images (down) are shown. Bars = 10 μm.

The reduced fertility of mature pollen grains had greatly attracted our interest; thus, we conducted further work to determine whether microspore nucleus underwent abnormal development by observing DAPI-stained spores at different developmental stages. The typical and normal process of microspore development was observed in the parent lines, and some normal microspore development could also be found in the *Brassica* allohexaploids (**Figures 2A–O**). Meiosis of PMCs resulted in a tetrad enclosed within a thick callose wall (**Figures 2A, F, K**). Microspores freely released from the tetrad (**Figures 2B, G, L**) gradually increased in size and became polarized, with the nucleus displaced to a future germ cell pole (**Figures 2C, H, M**). Then, a curved cell plate separated the two unequal daughter cells (**Figures 2D, I, N**). The smaller generative cells, which had nuclei stained intensely with DAPI, underwent pollen mitosis to generate two sperm cells (**Figures 2E, J, O**). However, some aberrant cell developments were observed at the tetrad stage, with various numbers of nuclei in the *Brassica* allohexaploids (**Figures 2P, Q, U, V**). Both *B. rapa* and *B. carinata* had 100% normal tetrads composed of four equally sized microspores, but this proportion was reduced to 83.5% at the tetrad stage in the *Brassica* allohexaploids (**Supplementary Figures S3A**). Parts of the tetrads (14.7%) were severely abnormal, with highly variable numbers of spores from monad to triad (**Figures 2P, Q, U, V**). Even relatively normal-looking tetrads in the *Brassica* allohexaploids gave rise to microspores undergoing defective asymmetric cell division, which might account for the high proportion of abnormal pollen grains (34.6%) with equal nuclei (**Figure 2R**) and abnormal nuclei (**Figure 2W**) observed at the early bicellular stage (**Supplementary Figure S3B**). *B. rapa* and *B. carinata* had almost all normal tricellular pollen, while the *Brassica* allohexaploid plants had 65.5% normal pollen, and the rest showed a mixture of phenotypes, including binucleate and uninucleate pollen grain (**Figures 2S, T, X, Y**; **Supplementary Figure S3C**). Taken together, these results clearly showed that the some of microspore development was defective in the synthetic *Brassica* allohexaploids.

### 3.4 Aberrant chromosome behavior during meiosis in the *Brassica* allohexaploids

**Figure 3:**
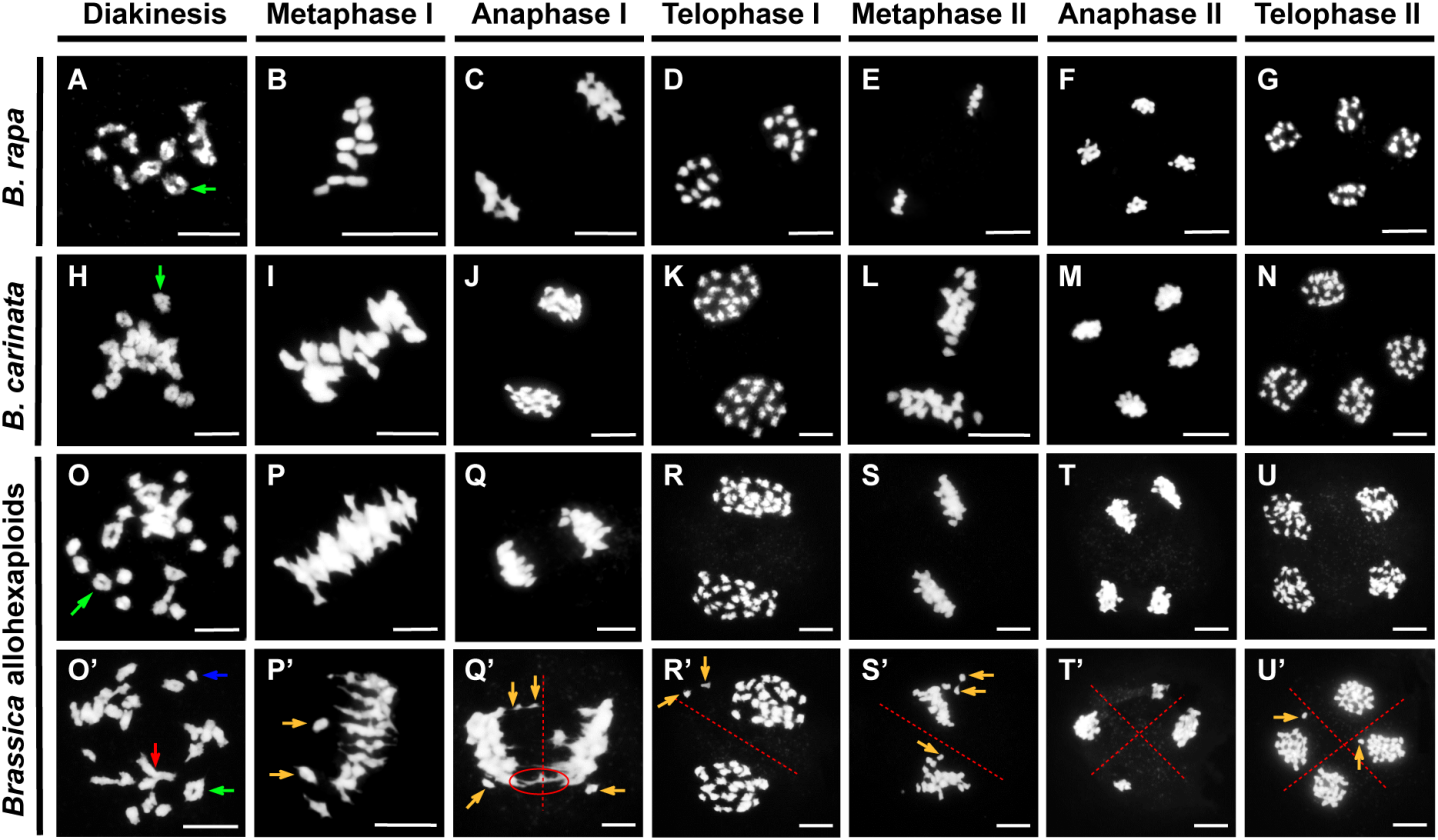
Meiotic chromosome behavior in *B. rapa*, *B. carinata* and *Brassica* allohexaploids. Normal meiosis processes in *B. rapa* **(A–G)**, *B. carinata* **(H–N)** and *Brassica* allohexaploids **(O–U)**. **(O’–U’)** Abnormal chromosome behavior of pollen mother cells (PMCs) in the *Brassica* allohexaploid plants. The green arrow indicates a normal bivalent; the blue arrow indicates a univalent; the red arrow indicates a multivalent; and the brown arrow indicates a lagging chromosome. The chromosome bridge is indicated with a red ellipse. Bars = 10 µm.

The majority of synthetic polyploids exhibit meiosis aberration, which might lead to abnormalities in pollen development (Tian et al., 2010; Gaebelein et al., 2019). Thus, we assessed chromosome behavior during meiosis in the PMCs of the *Brassica* allohexaploid plants. Throughout meiosis, some normal chromosome behavior was detected in the *Brassica* allohexaploids (**Figures 3O–U**), as was generally observed in *B. rapa* and *B. carinata* (**Figures 3A–N**). These all normal chromosomes were predominantly condensed into bivalents at diakinesis (**Figures 3A, H, O**), and at metaphase I, the bivalents were orderly aligned along the metaphase plate (**Figures 3B, I, P**). The homologous chromosomes were evenly separated through anaphase I and telophase I (**Figures 3C, D, J, K, Q, R**). Then, two groups of condensed sister chromosomes were arranged at the equatorial plate during metaphase II (**Figures 3E, L, S**). Finally, chromatids separated to each spindle pole at anaphase II and formed tetrads at telophase II (**Figures 3F, G, M, N, T, U**).

Normal chromosome behavior could be observed in the *Brassica* allohexaploid plants, and bivalents were detected with high frequency, but univalents and multivalents were also frequently observed in PMCs from the *Brassica* allohexaploids at diakinesis (**Figures 3O, O’**). At metaphase I, 85.6% of PMCs carried certain chromosomes detached from the equatorial plate with lagging chromosomes (**Figures 3P’**; **Supplementary Figure S4**). Subsequently, meiotic chromosomes were segregated, and chromosome bridges and unequal segregation events occurred in 72.4% of PMCs at anaphase I and in 36.9% of PMCs at anaphase II (**Figures 3Q’, R’**; **Supplementary Figure S4**). At metaphase II, 86.5% PMCs were observed with lagging chromosomes, which finally led to unbalanced gametes with unequal chromosome numbers in each microspore after telophase II (**Figures 3S’, U’**; **Supplementary Figure S4**). These observations suggested that the development of PMCs had severe defects in the *Brassica* allohexaploids.

### 3.5 Abnormal bipolar spindle during meiosis in the *Brassica* allohexaploids

**Figure 4:**
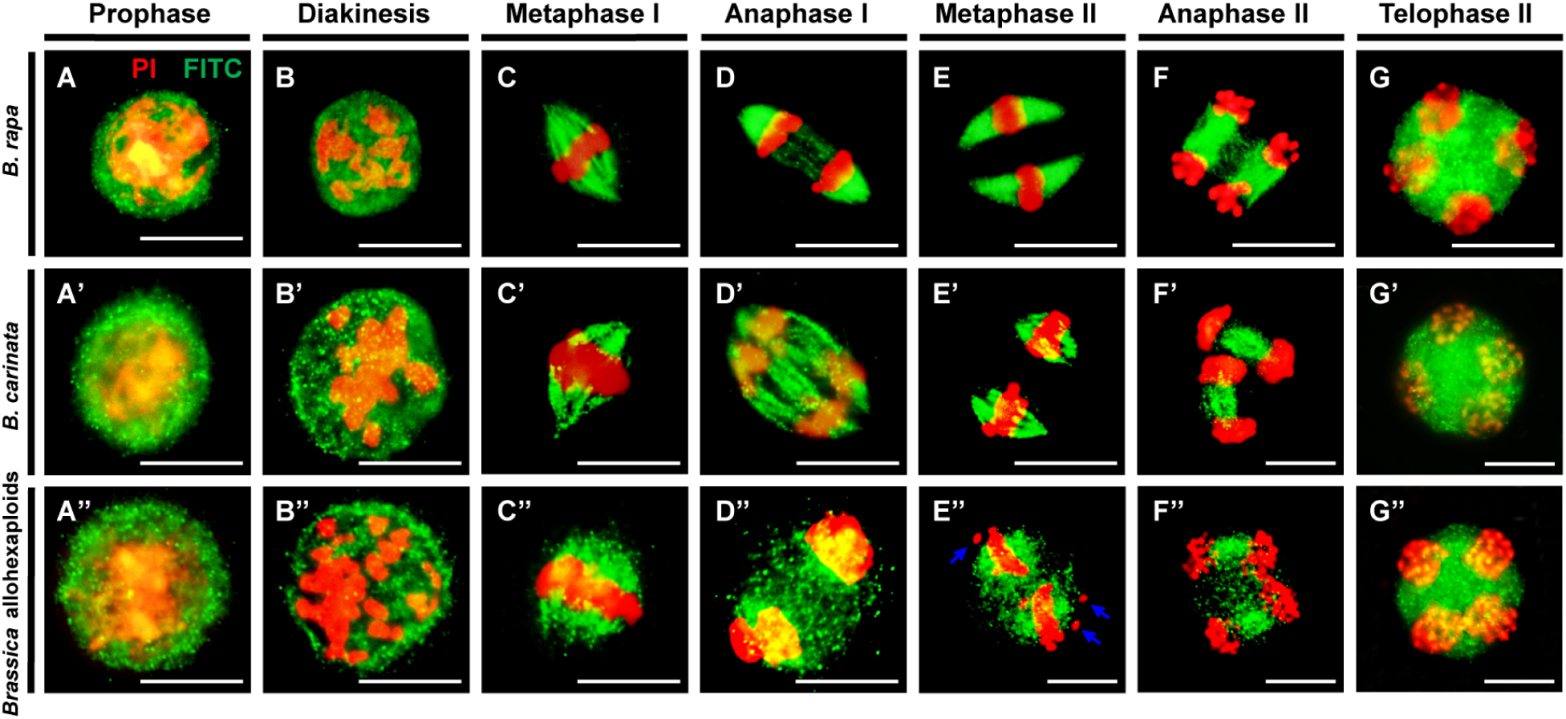
Spindle organization during meiosis in *B. rapa*, *B. carinata* and *Brassica* allohexaploids. **(A–G)** Normal spindle organization and chromosome behavior observed in pollen mother cells (PMCs) of *B rapa*. (**A’–G’)** Normal spindle organization and chromosome behavior observed in PMCs of *B carinata*. **(A”–G”)** Abnormal spindle organization and chromosome behavior observed in PMCs of the *Brassica* allohexaploids. Microtubules and chromosomes are colored in green and red, respectively. Blue arrows indicate lagging chromosomes. Bars = 10 µm.

In order to observe the microtubule dynamics of the newly synthesized *Brassica* allohexaploids during meiosis, the microtubule dynamics with separating chromosomes during meiosis were studied by β-tubulin immunostaining. Balanced chromosome segregation and complete meiotic cytokinesis were observed in *B. rapa* and *B. carinata* (**Figure 4**). During pachytene and diakinesis, condensed chromosomes and some microtubules appeared as intense foci around the perinuclear zone (**Figures 4A, B, A’, B’**). At metaphase I, microtubules were organized into the spindle structure and then attached to the kinetochores, forming a typical bipolar fusiform configuration at the metaphase plate (**Figures 4C, C’**). Then, the spindle pulled each group of homologous chromosomes to the polar side of the cell at anaphase I (**Figures 4D, D’**). During meiosis II, two pairs of spindles were constructed at metaphase II (**Figures 4E, E’**) and ensured the accurate disjunction of sister chromatids at anaphase II (**Figures 4F, F’**), consequently contributing to tetrad formation, where radial microtubule arrays were normally generated surrounding the nuclei at telophase II (**Figures 4G, G’**).

In contrast, the *Brassica* allohexaploids showed alterations in some microtubule dynamics. During the early and later stages of meiosis, the *Brassica* allohexaploids exhibited a microtubule distribution pattern similar to that in *B. rapa* and *B. carinata* (**Figures 4A”, B”**). However, there were some irregular spindles with punctate foci of signals indicating a failure of the typical bipolar spindle structure (**Figure 4C”**). Notably, some chromosomes were not attached to the meiotic spindles in the *Brassica* allohexaploids (**Figure 4E”**). Some microtubule fibers were more scattered at anaphase I, anaphase II and telophase II, which could not guarantee the accomplishment of successful and accurate chromosome segregation in the *Brassica* allohexaploids (**Figures 4D”, F”, G”**). The abnormal microtubules were counted as 6.61%, 27.84% and 61.52% in *B. rapa*, *B. carinata* and *Brassica* allohexaploids, respectively (**Supplementary Figure S5**). These results demonstrated that impaired bipolar spindle during meiosis may contribute to low pollen fertility in the newly synthesized *Brassica* allohexaploids.

### 3.6 Chromosome set of B genome segregated equally during meiosis in the *Brassica* allohexaploids

Chromosomes must first recognize their homologous partners and then pair with them during early meiotic prophase I to ensure accurate chromosome segregation (Xiong et al., 2021). Hence, chromosome segregation defects generally exist in plants with chromosome pairing problems. To reveal the interactions between the three genomes resulting from hybridization and genome doubling in the *Brassica* allohexaploid plants, GISH analysis was performed with the B genome probes from the B genome of *B. nigra*.

**Figure 5:**
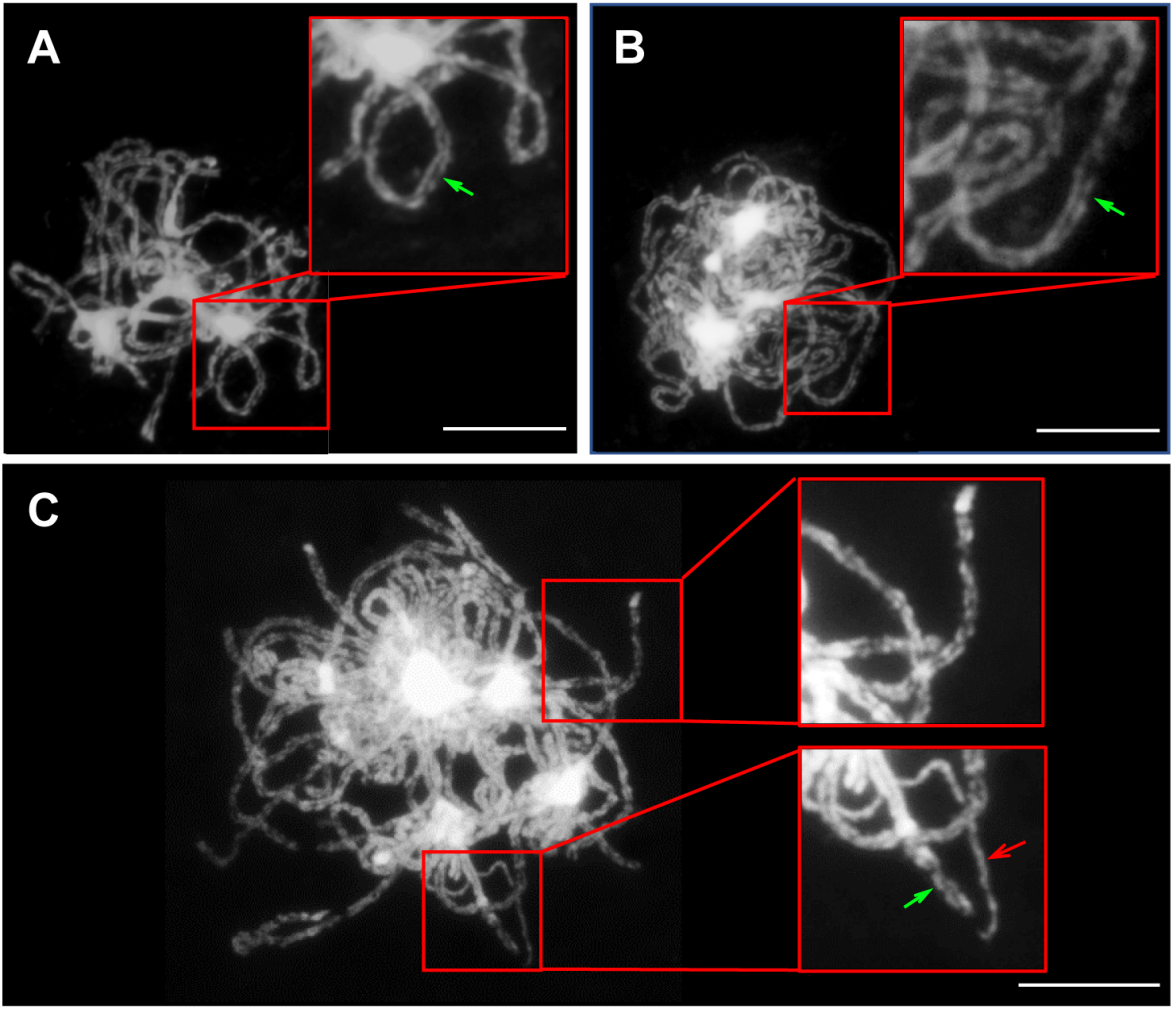
Pairing and synapsis of homologous or homoeologous chromosomes at pachytene. Fully synapsed and paired chromosomes in *B. rapa* **(A)** and *B. carinata* **(B)**; in contrast, partially synapsed and pairing chromosomes at pachytene in the *Brassica* allohexaploids **(C)**. Green arrows indicate normal synapsed and paired chromosomes, while red arrows indicate the non-synapsed and unpaired chromosomes. Bars = 10 µm.

**Figure 6:**
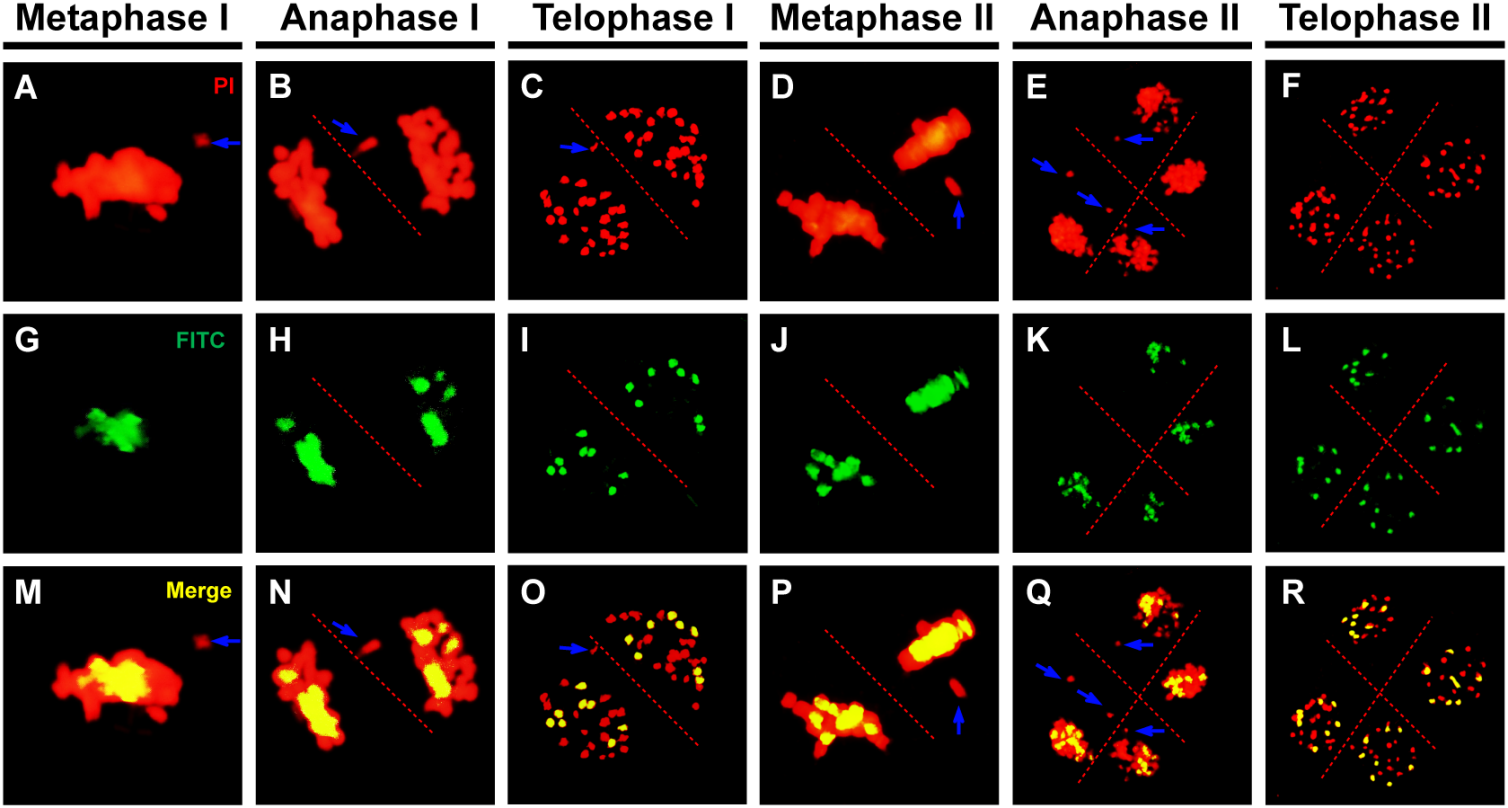
Genomic *in situ* hybridization (GISH) analysis of the *Brassica* allohexaploids during meiosis. **(A–F)** Chromosomes stained with propidium iodide (PI, red). **(G–L)** B genome chromosomes labeled by fluorescein isothiocyanate (FITC, green). **(M–R)** Merged overlay of the two signals (yellow). Blue arrows indicate lagging chromosomes from the A or C genome. Bars = 10 µm.

Homologous chromosomes were closely aligned at the pachytene in both *B. rapa* and *B. carinata*, indicating complete synapsis and pairing (**Figures 5A, B**). Although almost all of the chromosomes could complete synapsis and pairing in the *Brassica* allohexaploids, the existence of unpaired chromosomes in most PMCs of the *Brassica* allohexaploids suggested the occurrence of incomplete pairing and synapsis (**Figure 5C**). Meanwhile, GISH results showed that all of the chromosomes of the B genome were cohesively placed on the equatorial plate at metaphase I (**Figures 6A, G, M**) and metaphase II (**Figures 6D, J, P**), while lagging chromosomes were stained red without the FITC label, indicating that the lagging chromosomes were from the A and C genomes rather than the B genome. Moreover, the lagging chromosomes and chromosome bridges also existed without B genome-labeled chromosomes at anaphase I (**Figures 6B, H, N**) and anaphase II (**Figures 6E, K, Q**), demonstrating no B genome chromosome loss in the *Brassica* allohexaploids. In addition, the chromosome sets of the B genome were regularly distributed in dyad PMCs, with eight chromosomes in each pole at telophase I (**Figures 6C, I, O**), and could be equally segregated into tetrads at telophase II (**Figures 6F, L, R**). Therefore, these results indicated that B genome was less prone to missegregation compared to A and C genome during meiosis in the *Brassica* allohexaploids.

### 3.7 Genes related to meiotic chromosome segregation were mostly downregulated in the *Brassica* allohexaploids

**Figure 7:**
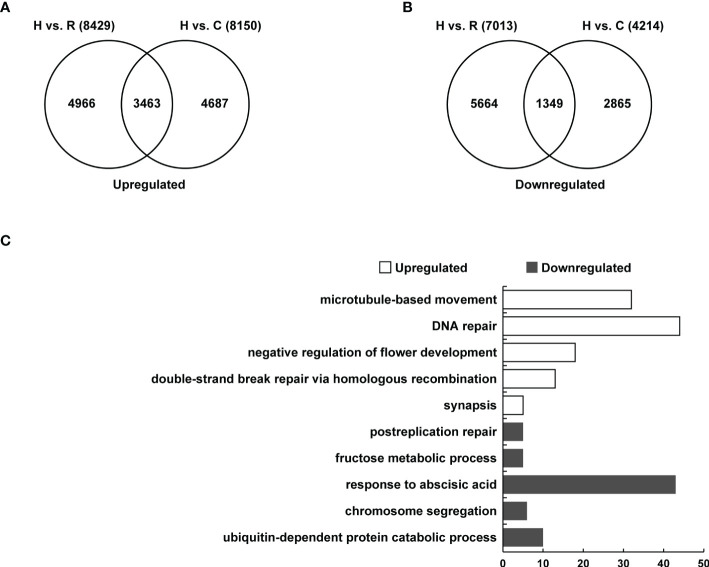
Transcriptome analysis of flower buds at meiosis in the *Brassica* allohexaploids. **(A)** Venn diagram showing the overlap of upregulated genes between *Brassica* allohexaploids vs. *B. rapa* and *Brassica* allohexaploids vs. *B. carinata*. **(B)** Venn diagram showing the overlap of downregulated genes between *Brassica* allohexaploids vs. *B. rapa* and *Brassica* allohexaploids vs. *B. carinata*. **(C)** GO classification of the overlapping genes upregulated and downregulated between *Brassica* allohexaploids vs. *B. rapa* and *Brassica* allohexaploids vs. *B. carinata*.

To determine the gene expression changes of floral buds in the synthesized *Brassica* allohexaploids, transcriptome analysis of the *Brassica* allohexaploids and their parents was performed using RNA-Seq. In the flower buds of the *Brassica* allohexaploids, 8249 genes were upregulated compared with *B. rapa* and 8150 genes were upregulated compared with *B. carinata* (**Figure 7A**). Venn diagram showed that there were 3463 genes upregulated in the flower buds of the *Brassica* allohexaploids compared to their parents (**Figure 7A**). The flower buds of the *Brassica* allohexaploids had 7013 downregulated genes compared with *B. rapa* and 4214 downregulated genes compared with *B. carinata* (**Figure 7B**). The 1349 genes were found to be downregulated in the flower buds of the *Brassica* allohexaploids as compared to their parents (**Figure 7B**). These findings demonstrated that the *Brassica* allohexaploids had more upregulated genes than downregulated genes compared with their parents. To explore the functional differences of DEGs among the flower buds between the *Brassica* allohexaploids and their parents, we focused on the significantly enriched GO Biological Process (BP) terms. In the flower buds of the *Brassica* allohexaploids, genes upregulated compared to their parents were mainly significantly enriched in microtubule-based movements (GO:0007018), DNA repair (GO:0006281), negative regulation of flower development (GO:0009910), double-strand break repair *via* homologous recombination (GO:0000724) and synapsis (GO:0007129), while genes downregulated compared to their parents were more enriched in postreplication repair (GO:0006301), fructose metabolic process (GO:0006000), response to abscisic acid (GO:0009737), chromosome segregation (GO:0007059) and ubiquitin-dependent protein catabolic process (GO:0006511)(**Figure 7C**).

## 4 Discussion

### 4.1 Defective microspore development in the *Brassica* allohexaploids

Morphological analysis showed that pollen fertility was 66.1%, on average, in *Brassica* allohexaploids, which was significantly reduced compared to that in the parental lines (**Figure 1**). Tapetum development in the corresponding *Brassica* allohexaploids was normal during anther development (**Supplementary Figure S2**). This meant that tapetum development became normal and could support the normal anther development of Brassica allohexaploid plants during early cytological diploidization. However, the development of microspore nucleus was partially abnormal in the *Brassica* allohexaploids. The percentage of abnormal microspore development increased from the tetrad stage to the tricellular stage (from 16.5% to about 34%; **Supplementary Figure S3**) in Brassica allohexaploids, indicating partially abnormal microspore development in nascent allohexaploids. Collectively, in addition to the typically reported meiotic defects in polyploids, these observations indicate the existence of defective microspore development in *Brassica* allohexaploids, which might be another result of meiotic defective or genetic exchange between different genomes or even a nucleo-cytoplasmic interaction (Comai, 2005; Geng et al., 2013; Yang et al., 2016). These results suggest that microspore abnormalities may affect the fertility of newly synthesized *Brassica* allohexaploids. The gene expression patterns synthesized *Brassica* allohexaploids and their parents were compared in this study, to further reveal the molecular mechanisms affecting pollen development. Enrichment of upregulated genes in the negative regulation of flower development may affect pollen fertility.

### 4.2 Abnormal meiotic chromosome behavior and spindle in the *Brassica* allohexaploids

Meiosis is the key biological process that underpins sexual reproduction (Osman et al., 2011). Thus, successful and accurate chromosome segregation during meiosis is significant for genetic stability during sexual reproduction, which might be a crucial challenge in polyploids consisting of more than two sets of chromosomes (Tian et al., 2010; Mwathi et al., 2017). For *Brassica* allotriploids, severely unstable meiosis occurred with variations in chromosome behavior, such as univalents and multivalents at diakinesis, lagging chromosomes at metaphase, anaphase and telophase, unequal segregation and chromosome bridges at anaphase in all PMCs (Yang et al., 2020). For the corresponding *Brassica* allohexaploids, stable chromosomal behavior was observed in some PMCs (**Figures 3O–U**). However, unstable meiosis still occurred in *Brassica* allohexaploid plants (**Figures 3O’–U’**), putatively resulting from unstable chromosome pairing and genetic recombination between homoeologous and non-homologous regions during early cytological diploidization in *Brassica* allohexaploids (Tian et al., 2010; Geng et al., 2013; Gupta et al., 2016; Zhou et al., 2016). The downregulation of *ASK1* suggested that chromosome segregation may have been shown to perform important roles in pollen fertility in the *Brassica* allohexaploids (Zhao et al., 2006). Moreover, the bipolar spindle is essentially required for chromosome movement and segregation during meiosis, and precise chromosome segregation is accomplished by the proper attachment of chromosomes to spindle microtubules *via* the kinetochore (Duro and Marston, 2015; Severson et al., 2016). This study demonstrated that no significant reduction of microtubule fibers occurred during meiosis in *Brassica* allohexaploids; however, incomplete spindle organization (**Figure 4**) could not provide enough force for accurate chromosome movements, thereby causing meiosis abnormalities (Hotta et al., 2012). Genes for chromosome segregation were downregulated may lead to reduced pollen fertility.

## 5 Conclusions

In the present work, we investigated the complete cytological process during male gamete formation in *Brassica* trigenomic allohexaploids *via* a cross between natural allotetraploid *B. carinata* and diploid *B. rapa* followed with chromosome doubling. In the newly synthesized *Brassica* allohexaploids, anther tapetum developed normally, while the microspore development was defective. In addition, chromosome behavior was mostly irregular, and the bipolar spindle during meiosis was partially abnormal in the *Brassica* allohexaploids. At the transcriptional level, the upregulation of genes related to the negative regulation of flower development and the downregulation of genes related to chromosome segregation may influence pollen fertility in the *Brassica* allohexaploids. Taken together, our results provide detailed cytological and transcriptomic insights into pollen development in the newly synthesized *Brassica* allohexaploids, which would be considered as a useful germplasm for *Brassica* polyploid breeding.

## Data availability statement

The data presented in the study are deposited in the NCBI, accession number GSE201456, GSE193368 and GSE185639.

## Author contributions

FW, GS and XW conceived, designed and instructed the study. BT, GC and YY bred the plant materials. ZX and XS assisted with material identification. XS, ZT and CJ performed the main experiments and data analysis, and wrote the manuscript. ZT, CJ and FW amended the manuscript. All authors contributed to the article and approved the submitted version.
