# Supplementary material for: Integrated cytological and transcriptomic analysis reveals insights into pollen fertility in newly synthetic Brassica allohexaploids
Source: Front Plant Sci. 2023 Jan 13;13:1096804. doi: 10.3389/fpls.2022.1096804 (PMC9880477; doi:10.3389/fpls.2022.1096804)
Supplement: Supplementary file 1 [file DataSheet_1.docx]

Supplementary Material


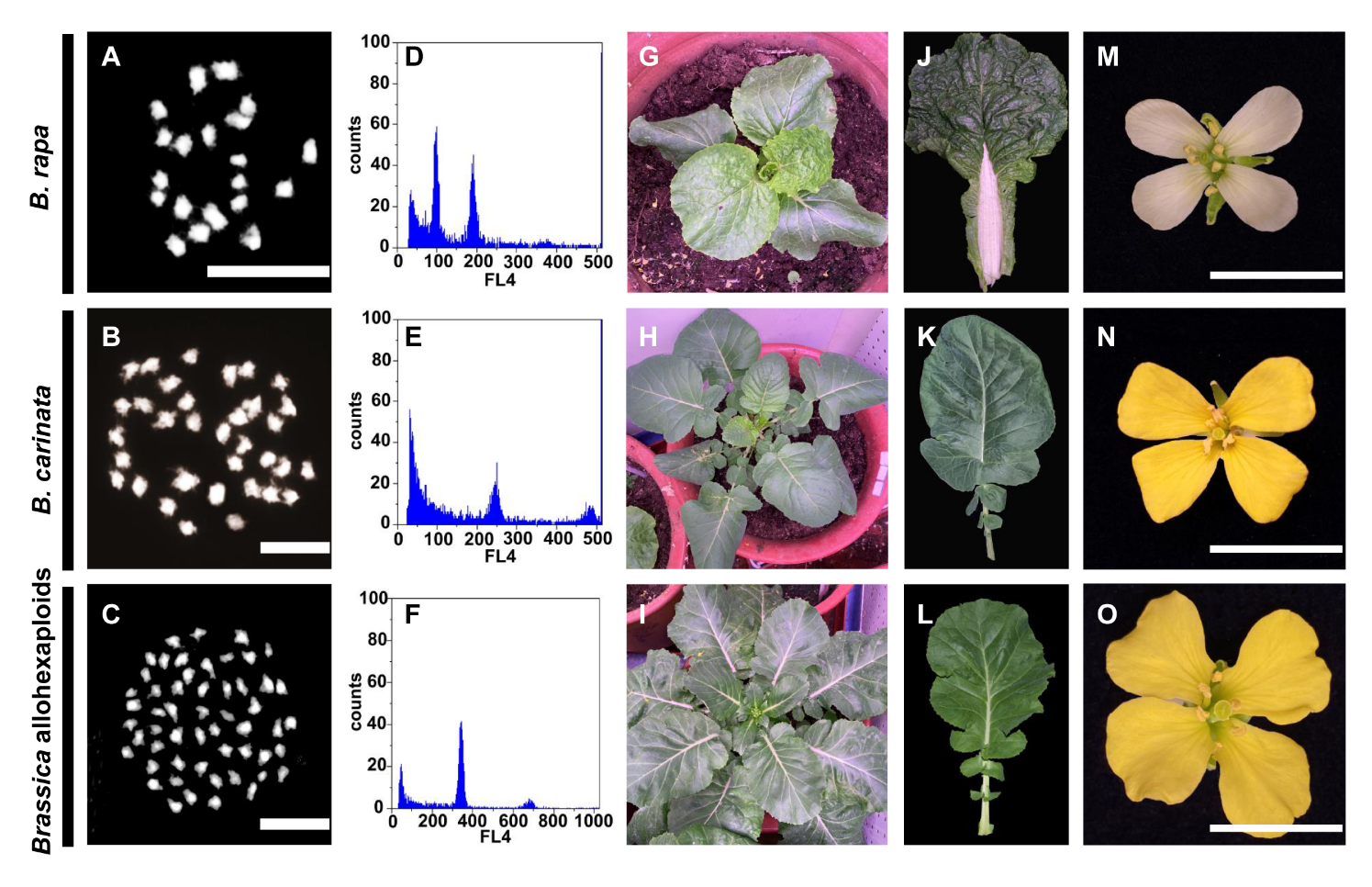


**Supplementary Figure S1.** Ploidy identification and morphological observation of *B. rapa*, *B. carinata* and *Brassica* allohexaploids. **(A–C)** Chromosome counting with 20 in *B. rapa*, 34 in *B. carinata* and 54 in *Brassica* allohexaploids. Bars = 10 μm. **(D–****F)** Flow cytometry of *B. rapa*, *B. carinata* and *Brassica* allohexaploids. **(G–I)** Plant morphology of *B.* *rapa*, *B.* *carinata* and *Brassica* allohexaploids. **(J–L)** Leaf morphology of *B.* *rapa*, *B.* *carinata* and *Brassica* allohexaploids. **(M–O)** Flower morphology of *B.* *rapa*, *B.* *carinata* and *Brassica* allohexaploids. Bars = 1 cm.


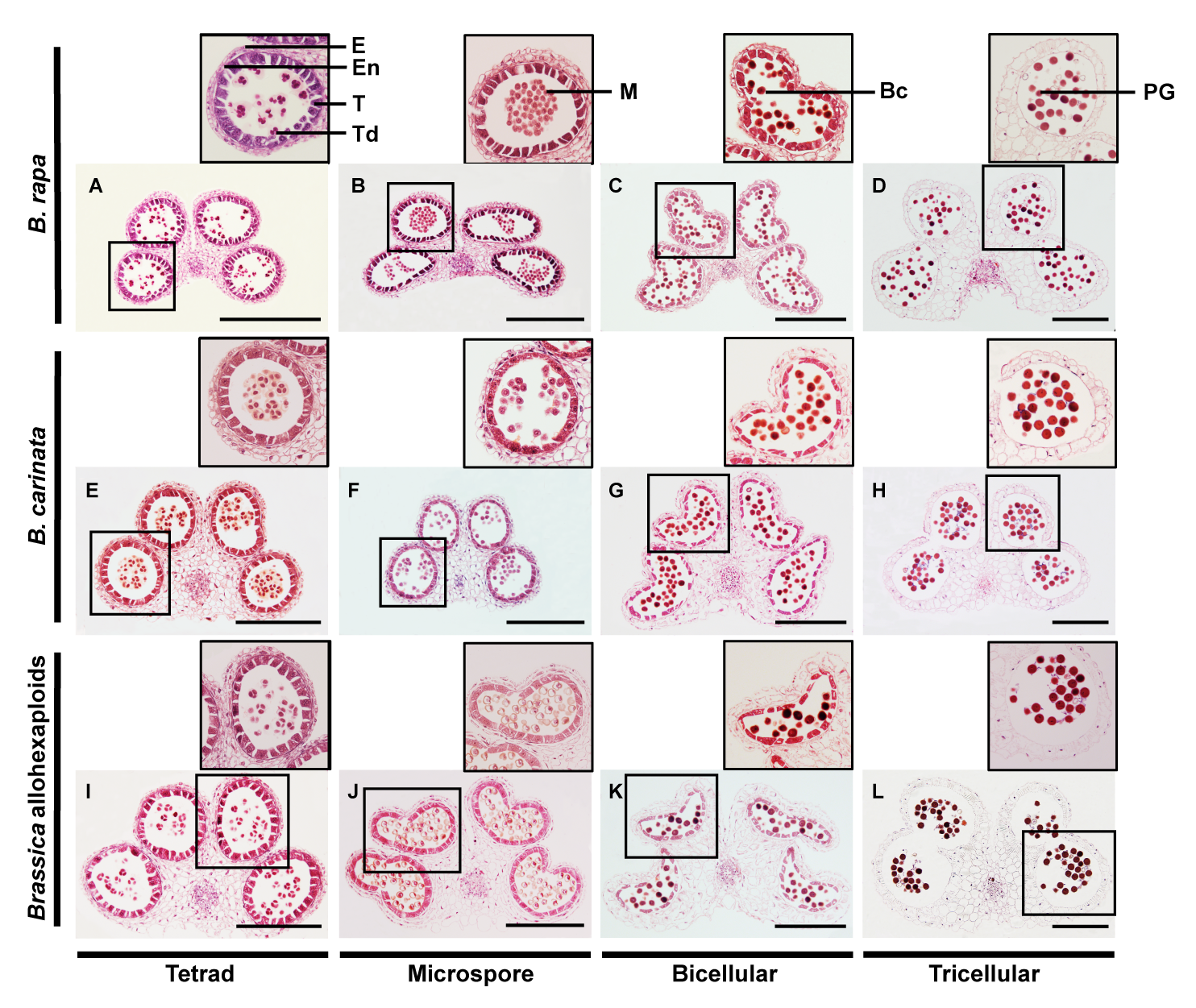


**Supplementary Figure S2.** Anther tapetum observation. **(A–D)** *B.* *rapa*, **(E–H)** *B.* *carinata* and **(I–L)** *Brassica* allohexaploids. (E) Epidermis; (En) Endothecium; (T) Tapetum; (Td) Tetrad; (Bc) Bicellular; (PG) Pollen grains. Bars = 200 µm.


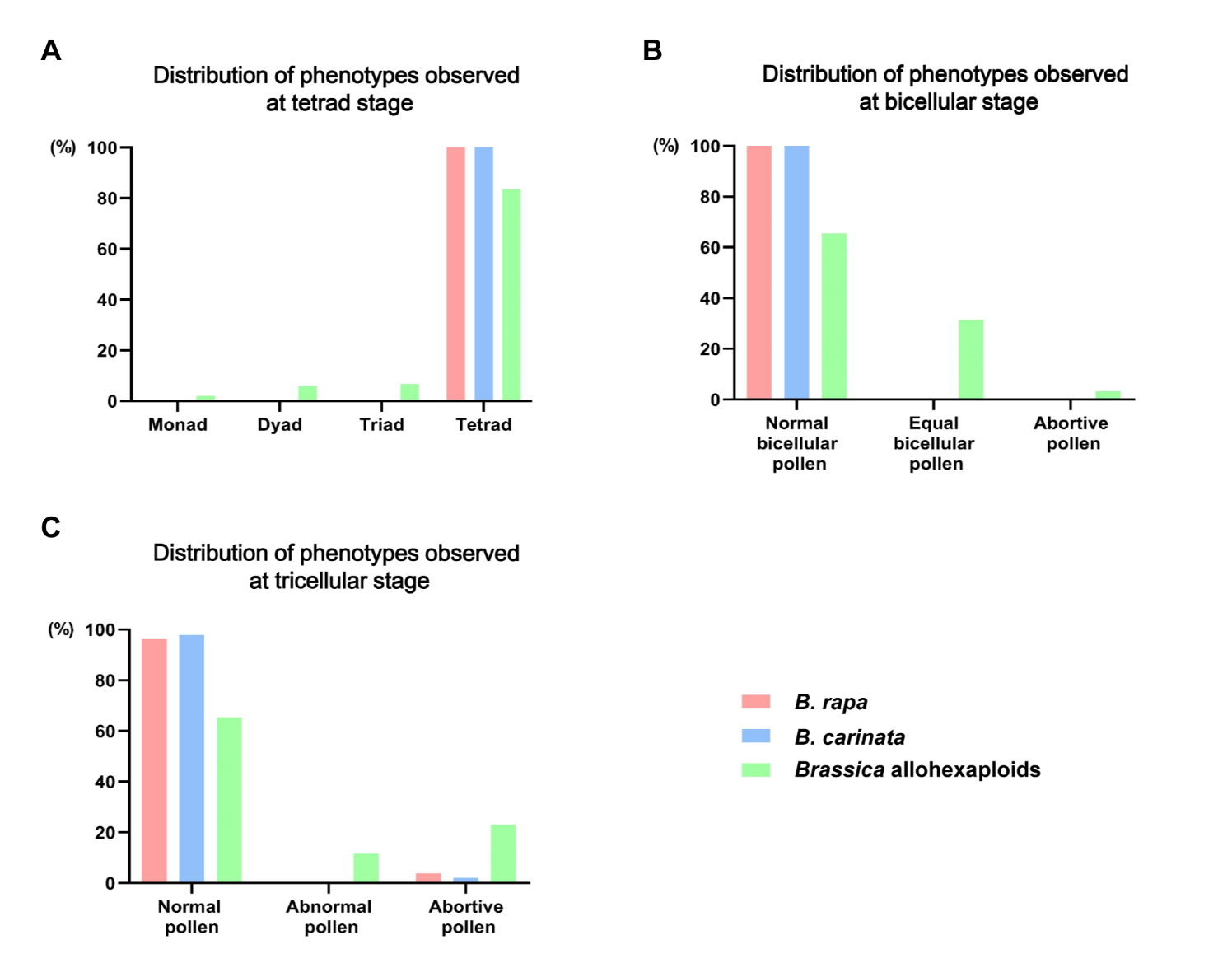


**Supplementary Figure S3.** The percentage of abnormal incidents during microspore development. **(A–C)** Distribution of microspore phenotypes observed in the tetrad, bicellular and tricellular stages of *B.* *rapa*, *B.* *carinata* and *Brassica* allohexaploids.


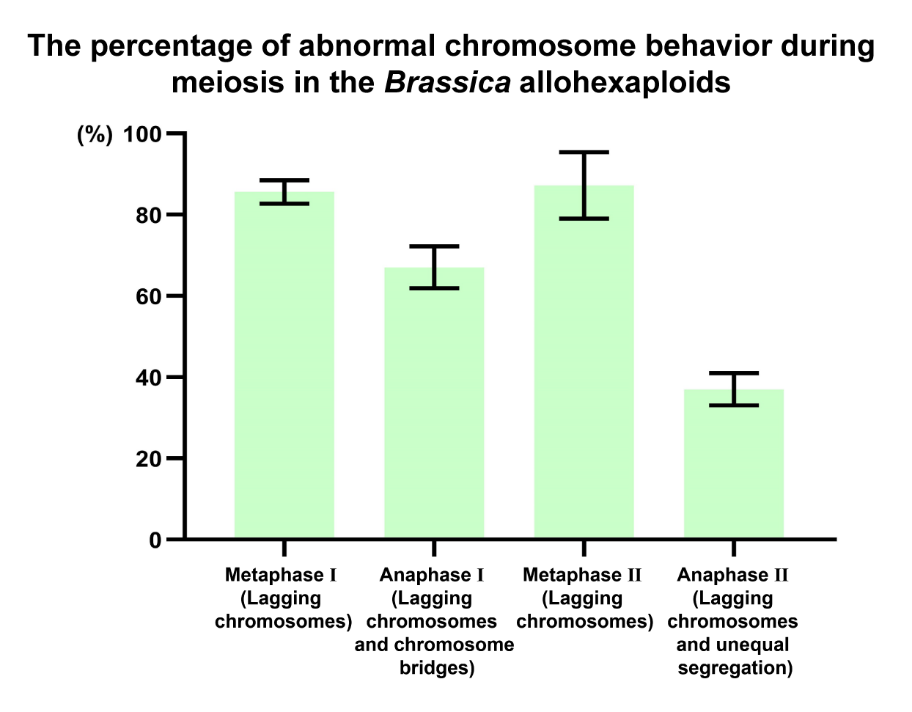


**Supplementary Figure S4.** The percentage of abnormal chromosome behavior during meiosis in the PMCs of *Brassica* allohexaploids. The mean values with standard deviation bar are shown in the figure.

**
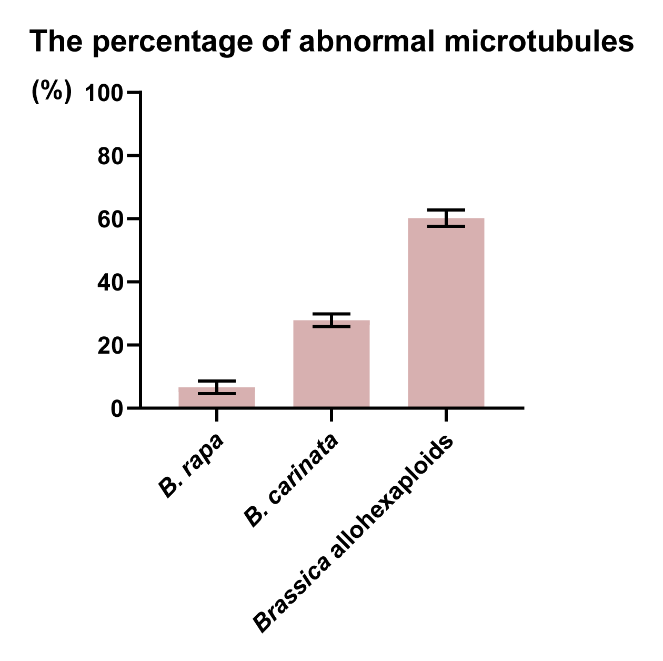
**

**Supplementary Figure S5.** The percentage of abnormal microtubules during meiosis in the *B. rapa*, *B. carinata* and *Brassica* allohexaploids.
